# Three-Dimensional Motion Analysis of the 2nd Cervical Spinous Process at End Range Cervical Rotation in Different Scapular Positions Using 3D Digitizer

**DOI:** 10.1155/2018/9835846

**Published:** 2018-08-26

**Authors:** Takahiro Otsudo, Kiyokazu Akasaka, Hiroshi Hattori, Yuki Hasebe, Akihiro Tamura, Toby Hall

**Affiliations:** ^1^School of Physical Therapy, Faculty of Health and Medical Care, Saitama Medical University, 981 Kawakado, Moroyama City, Iruma-Gun, Saitama 350-0496, Japan; ^2^Kawagoe Clinic, Saitama Medical University, 21-7 Wakitahonchyo, Kawagoe City, Saitama 350-1123, Japan; ^3^Department of Rehabilitation, Saitama Medical Center, Saitama Medical University, 1981 Kamoda, Kawagoe City, Saitama 350-8550, Japan; ^4^Sekishindo Hospital, 25-19 Wakitahonchyo, Kawagoe City, Saitama 350-1123, Japan; ^5^School of Physiotherapy and Exercise Science, Curtin University, Kent Street, Bentley, Perth, Western Australia 6102, Australia

## Abstract

**Objective:**

The study used a 3D digitizer to determine three-dimensional motion analysis of the 2nd cervical (C2) spinous process at end range cervical rotation with the scapula in different positions.

**Methods:**

30 healthy adults participated in this study. Different scapula positions were adopted bilaterally and positioned passively at normal resting, depression, adduction, and abduction. Under each scapula position, bilateral end range cervical rotation and displacement of the C2 spinous process were analyzed by a 3D digitizer.

**Results:**

Displacement of the C2 spinous process relative to the occiput was significantly correlated with range of cervical rotation under all scapular positions (p<0.05). However, there were no significant differences between end range cervical rotation and displacement of the C2 spinous process relative to the occiput in any scapular position.

**Conclusion:**

These results suggest that measurement of upper cervical mobility using the 3D digitizer is a reliable method that holds promise in the evaluation of people with cervical spine disorders.

## 1. Introduction

Neck pain has considerable impact on societal health, representing 14.6% of all musculoskeletal problems reported annually [1]. Assessment of active cervical spine movement is a routine part of examination of cervical spine disorders including neck pain [2]. Active cervical examination, however, incorporates movements of both upper and lower cervical segments simultaneously. Cervical rotation between the 1st cervical vertebra (C1) and the 2nd cervical vertebra (C2) accounts for approximately 50% of the total rotation in the cervical spine [3].

Impairments in alignment of the cervical spine and scapulae are commonly cited as possible sources of pain and dysfunction [4–6]. The scapulae and the cervical spine are anatomically linked through levator scapulae (LS) and trapezius (TR) muscles [7] among others. Changes in the alignment of the scapulae can potentially influence the biomechanics of cervical spine by altering tension in these muscles [8]. However, there is disagreement in the literature about the effects of scapula position on cervical movement and pain. For instance, passive scapular elevation significantly decreased neck pain and significantly increased cervical range of motion [9]. In contrast, another report found that scapula position did not influence range of cervical rotation [7]. Hence, it is unclear whether cervical rotation is influenced by different scapular positions or not.

When assessing range of upper cervical rotation, the cervical flexion rotation test (FRT) has been recommended [10]. In this test, the cervical spine is placed in end-range flexion to isolate rotation to the C1-2 motion segment. Therefore, the FRT may be a useful measure of upper cervical rotation in the clinical and research setting. However, the FRT may not be useful in evaluating the effect of scapular position on the upper cervical spine since the test is conducted in supine. On the other hand, three-dimensional motion of the spine and peripheral joints can be evaluated reliably and accurately by the 3D digitizer device. Cronbach's coefficient for intraobserver and interobserver reliability of the 3D digitizer was 0.92 for both, which indicates high internal consistency [11]. Furthermore, reproducibility of measuring bone or spatial landmarks using the 3D digitizer device is high and is an easy-to-operate apparatus [12–14].

The purpose of this study was to investigate the effect of different scapular positions on two parameters, namely, range of cervical rotation as well as displacement between the occiput and C2 spinous process at end range cervical rotation (C2 displacement) using the 3D digitizer device.

We hypothesized that (1) range of C2 displacement would correlate with range of cervical rotation and that (2) range of C2 displacement and range of cervical rotation were both influenced by different scapular positions.

## 2. Material and Method

### 2.1. Subjects

Thirty healthy participants were recruited and interviewed about their medical history; none had current neck pain, systemic arthritis, systemic bone disease, neuromuscular disease, or any history of cervical surgery. All participants agreed to sign an informed consent declaration. The 30 participants were 15 males and 15 females of mean age 21.2 ± 0.8 years, mean height 166.4 ± 8.8 cm, and mean body mass 60.2 ± 9.7 kg. This study followed the Declaration of Helsinki and was approved by the Ethics Committee at the Saitama Medical University, Saitama, Japan (no. 166).

### 2.2. Experimental Process

**Figure 1:**
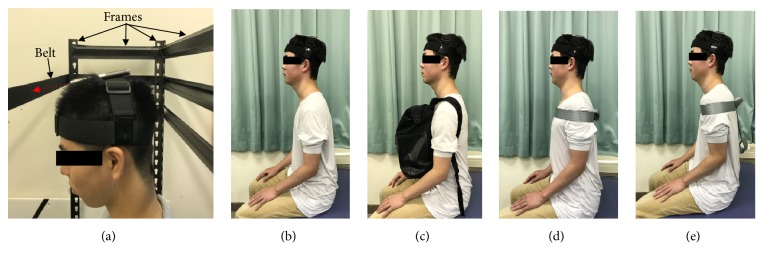
Set-up for measurement of end range cervical rotation and scapula position. (a) A laser pointer was fixed to the head with strap and the light (red arrow) centered on a band (width; 50 mm) set within an adjustable height frame to facilitate cervical rotation and minimize flexion and extension. (b) Resting position. (c) Scapular depression was standardized by carrying a backpack on the chest containing a 6 kg weight. (d) Scapular adduction was standardized with a nonelastic belt securely fastened around the chest wall with both scapulae retracted. (e) Scapular abduction was standardized with a nonelastic belt securely fastened around the chest wall with both scapulae protracted.

In this study, with the subject seated on a bench, scapular position was bilaterally and passively set at (1) resting position, (2) depression, (3) adduction, and (4) abduction, with a neutral glenohumeral joint position. Subjects were surrounded by steel frames (Tactix™ Steel shelving Unit). Under the resting condition, subjects were in their normal relaxed seated position. Scapular depression was induced by carrying a backpack on the subject's chest containing a 6 kg weight. Scapulae adduction was achieved by a nonelastic belt securely fastened around the chest wall with both shoulder girdles extended while scapulae abduction was achieved with the same belt with both shoulder girdles flexed. A laser pointer (RX-4N, SAKURA COLOR PRODUCT Corp., Japan) was strapped to the subject's head (Head strap GoPro Corp., Japan). Subjects were required to maintain the laser light in the center of a horizontal band (width; 50 mm) which was set within a height-adjustable frame. The purpose of the laser light was to enable cervical rotation without flexion or extension (Figure 1).

The C2 spinous process, mastoid processes, and acromial angles were identified by palpation and subsequently scanned using the 3D digitizer device (Microscribe® G2X, Revware Inc., USA). Measurements were taken by manually palpating the specific bony landmarks (resolution; 1,000 *μ*m), with the data being directly entered to modelling software (Rhinoceros Ver.5.0 Robert McNeel & Associates., USA) and presented graphically. As a pilot to determine accuracy and reliability of the 3D digitizer device, we analyzed 12 lengths and angles of 3 standardized accurately manufactured cubes which were guaranteed as 10.0 × 10.0 × 10.0 [mm] (ATC-01607, Artec Co., Ltd.). As a result of this pilot study, ICC (1.1), ICC (2.1), 95%CI, and SEM (Standard Error of Measurement = SD (d) × (1-ICC) 1/2) for measuring the cube's lengths were as follows; ICC (1.2) = 0.99, 95%CI = 0.96-0.99, and SEM = 0.02 [mm], and ICC (2.1) = 0.91, 95%CI = 0.72-0.98, and SEM = 0.20 [mm]. ICC (1.2), ICC (2.1), 95%CI, and SEM for measuring the cube's angles were as follows: ICC (1.2) = 0.99, 95%CI = 0.96-0.99, and SEM = 0.14 [degrees], and ICC (2.1) = 0.99, 95%CI = 0.70-0.98, and SEM = 0.26 [degrees].

We verified the reliability of the measurement of the 3D digitizer by measuring the distance between the right and left mastoid process of the occipital bone in 30 subjects. As a result, the distance between the right and left mastoid process of the occipital bone [mm], ICC (1.2), 95%CI, F value, and SEM [mm] were 121.3 ± 10.3 [mm], 0.96, 0.93-0.98, 79.48, and 0.11 [mm], respectively. We also analyzed the ICC (1.2), 95%CI, F value, and SEM for the distance between the C2 spinous process and the line between the 2 mastoid processes in order to confirm the absence of cervical flexion or extension at end range cervical rotation. The results were ICC (1.2) = 0.89, 95%CI = 0.82-0.94, F value = 26.12, and SEM = 2.01 [mm], respectively.

**Figure 2:**
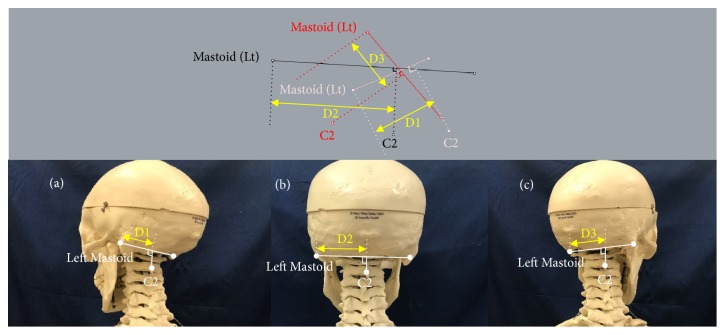
Analysis of C2 displacement at end range cervical rotation. The diagram in the upper section presents the data from one of the subjects analyzed with 3D coordinates consisting of both mastoid processes, C2 spinous process, and additional lines constructed by the modelling software. C2 displacement was calculated as the change in distance between the left mastoid process and the point of intersection from a perpendicular line drawn from the C2 spinous process to a line drawn between both mastoid processes, comparing the neutral position and each end range cervical rotation position. D1: C2 displacement at end range cervical left rotation. D2: C2 displacement at neutral position. D3: C2 displacement at end range cervical right rotation.

Range of cervical rotation to each side was determined by the vector from the left to right mastoid process related to the vector from left to right acromial angle. C2 displacement was calculated as the change in distance between the left mastoid process and the point of intersection from a perpendicular line drawn from the C2 spinous process to a line drawn between both mastoid processes, comparing the neutral position and each end range cervical rotation position (Figure 2). C2 displacement represents the change in distance between the occiput and C2 spinous process as a result of cervical rotation. Although C2 displacement cannot be directly converted into angular motion, C2 displacement is directly representative of atlantooccipital (C0-1) and/or atlantoaxial (C1-2) joint motion during cervical rotation.

Muscle hardness served as a proxy for muscle tension and was determined for the levator scapulae (LS-hardness) and trapezius (TR-hardness) muscles by the muscle hardness tester (NEUTONE TDM-N1 TRYALL Corp., Japan). This was used to determine whether muscle hardness was influenced by end range cervical rotation and C2 displacement or not. LS-hardness was measured bilaterally at the midpoint between the C2 spinous process and the medial aspect of the spine of scapula. TR-hardness was measured bilaterally at the midpoint between the C7 spinous process and the acromial angle.

### 2.3. Statistical Analysis

All data were analyzed using SPSS Statistics (version 22.0). Correlations between C2 displacement, LS-hardness, and TR-hardness at each scapular position were calculated using Spearman correlation. Repeated-measures ANOVA with Dunnett's post hoc test were used to compare range of cervical rotation and C2 displacement with the scapula in resting position with that at 3 different scapular positions (depression, adduction, and abduction). Significant differences were set at a level of 0.05.

## 3. Results

Bilateral range of cervical rotation was significantly correlated with C2 displacement relative to the occiput in all scapula positions (Table 1). These results indicated that C2 displacement was correlated with the magnitude of upper cervical rotation regardless of the four scapular positions: rest, depression, adduction, and abduction. Despite these findings, comparisons of mean range of cervical rotation and mean C2 displacement relative to the occiput were not significantly different in four scapular positions: rest, depression, adduction, and abduction (Table 2).

Muscle hardness of the right LS and left and right TR with the scapulae in resting position were correlated with range of right cervical rotation. Right LS-hardness with the scapula in resting position and bilateral LS-hardness and left TR-hardness with the scapulae in adduction were correlated with range of left cervical rotation.

## 4. Discussion

The results of this study support our hypothesis that C2 displacement with the scapulae in resting position correlated with range of cervical rotation as we hypothesized firstly. These results are consistent with a previous study that reported that range recorded during the FRT was correlated with total range of cervical rotation [15]. Interestingly, the current study found positive correlations between C2 displacement and range of cervical rotation in all scapular positions. Hence it was considered that upper cervical and whole cervical rotation mobility were not influenced by scapular position at least in healthy people.

According to previous studies, it has been reported that scapular depression may contribute to prolonged compressive loading of the cervical spine through the cervicoscapular muscles such as upper TR and LS [16]. Similarly, it has been reported that increased LS stiffness might contribute to compressive load and shear force on the cervical spine during active neck movement [17] and repetitive stress has the potential to cause cumulative microtrauma to tissues in the cervical region, thereby inducing neck pain and limiting neck range of motion [16, 18]. However, these comments were expressed as an opinion in the discussion of a single case report or for patients with widespread neck pain. We originally hypothesized that greater tension of the LS and TR would inversely affect cervical rotation. Our results regarding muscle hardness, which served as a proxy for muscle tension, showed that right LS-hardness and bilateral TR-hardness with the scapulae in a resting position were significantly and negatively correlated with range of right cervical rotation. Additionally, right LS-hardness with the scapulae in resting position, bilateral LS-hardness, and left TR-hardness with the scapulae in adduction were also significantly and negatively correlated with range of left cervical rotation. Interestingly, the results for muscle hardness were not consistent for the right and left in terms of C2 displacement and range of cervical rotation.

It has been proposed that scapular depression might be a contributing factor in neck pain [19], and scapular postural correction strategies have been advocated as a treatment strategy for patients with neck pain with altered scapular orientation [20, 21]. Previously it has been demonstrated that in healthy subject's cervical rotation range increased by up to 11° when support was provided under the subject's arms elevating their scapulae [7]. In general, it was assumed that supporting under the arms and elevating the scapulae increased end range cervical rotation by reducing passive tension of the upper TR and LS [9]. In those studies, a digital inclinometer was used to measure total cervical rotation [7, 9], and no attempt was made to assess rotation of the upper cervical spine. The current study did not compare the effect of a supported arm/scapulae position with other scapular positions (depression, adduction, and abduction) on range of cervical rotation; therefore we cannot determine whether scapula depression has an effect on cervical rotation range by altering LS or TR muscle stiffness length or not.

Clinically, upper cervical rotation is impaired in people with cervicogenic headache (CGH) [22]. The lateral and median C1-2 joints have synovial membranes, fibrous capsules, and ligament, respectively. In healthy people, C1-2 rotation is mainly restricted by the alar ligaments, with a minor contribution from the accessory atlantoaxial ligaments, as well as obliquus capitis inferior, rectus capitis posterior major, splenius capitis, and sternocleidomastoid muscles [23]. In order to increase C1-2 joint movement in CGH management, physical therapy has not focused on scapular position but instead recommendations have been made regarding improving cervical function directly through mobilization with cervical movement [24], suboccipital muscles release [25], or low-load craniocervical flexion exercise [26]. The current study's results also support these physical therapies for people with CGH because there were no significant differences in range of cervical rotation and displacement of C2 spinous process relative to the occiput in any scapular position.

Several limitations need to be noted with regard to the study findings. Firstly, C2 displacement in mm cannot be directly related to the angle of rotation in degrees. Secondly, subjects were all healthy, so it is not possible to conclude whether scapula position influences upper cervical or whole cervical rotation in people with neck pain or specific disorders such as CGH. Thirdly, the relationship is unclear between displacement of C2 and displacement of other cervical spinous processes at end range cervical rotation in different scapular positions. Further studies should focus on C2 displacement relative to the occiput at end range cervical rotation using the 3D digitizer for the patients with neck pain, CGH, and other associated disorders.

## 5. Conclusions

In this study, three-dimensional motion analysis of the C2 spinous process at end range cervical rotation in different scapular positions was assessed using the 3D digitizer device. C2 displacement relative to the occiput was significantly correlated with range of cervical rotation under all scapular positions. Despite this finding, there were no significant differences in range of cervical rotation and displacement of C2 spinous process relative to the occiput in any scapular position. These results suggest that measurement of upper cervical mobility using the 3D digitizer is a reliable method that holds promise in the evaluation of people with cervical spine disorders. Future studies should verify the measurement of 3D digitizer determined C2 displacement before acceptance of this measurement method in patients with dysfunction in the upper cervical spine.

## Figures and Tables

**Table 1:** Spearman's correlation coefficient of C2 displacement, levator scapulae, and trapezius muscle hardness related to end range cervical rotation, n = 30.

Scapula position	Rest		Depression		Adduction		Abduction	
Direction of rotation	R	L	R	L	R	L	R	L
C2 displacement	0.44*∗*	0.46*∗*	0.39*∗*	0.50*∗*	0.46*∗*	0.49*∗*	0.39*∗*	0.41*∗*
R LS-hardness	−0.37*∗*	−0.36*∗*	−0.18	−0.21	−0.21	−0.38*∗*	−0.12	−0.21
L LS-hardness	−0.28	−0.20	−0.13	−0.22	−0.25	−0.41*∗*	−0.27	−0.15
R TR-hardness	−0.43*∗*	−0.21	−0.21	−0.08	−0.07	−0.13	−0.01	−0.14
L TR-hardness	−0.41*∗*	−0.29	−0.09	−0.14	−0.30	−0.45*∗*	−0.22	−0.18

*∗*P<0.05, R: right, L: left, LS: levator scapulae muscle, and TR: trapezius muscle.

**Table 2:** End range cervical rotation and C2 displacement in different scapula positions, n =30.

Scapula position	Rest	Depression	Adduction	Abduction
End range cervical right rotation	70.1 ± 7.9	67.9 ± 6.7	70.0 ± 5.8	69.8 ± 7.1
End range cervical left rotation	69.7 ± 8.2	66.7 ± 8.6	70.2 ± 9.1	68.9 ± 9.5
C2 displacement at end range cervical right rotation	18.9 ± 7.2	17.1 ± 7.2	19.1 ± 9.6	18.3 ± 7.0
C2 displacement at end range cervical left rotation	24.8 ± 6.7	27.1 ± 7.5	24.6 ± 10.9	25.6 ± 9.7

End range cervical rotation to the right and left measured in degrees.

C2 displacement at end range cervical rotation to the right and left measured in mm.

There were no significant differences of both end range cervical rotation and C2 displacement in all different scapular positions.

## Data Availability

The data used to support the findings of this study are available from the corresponding author upon request.

## References

[1] Côté P., Cassidy J. D., Carroll L. J., Kristman V. (2004). The annual incidence and course of neck pain in the general population: a population-based cohort study. *PAIN*.

[2] Maitland G., Hengeveld E., Banks K., English K. (2001). *Maitlands vertebral manipulation*.

[3] Bland J., Singer K., Giles L. G. F. (1998). Anatomy and pathology of the cervical spine. *Clinical Anatomy and Management of Cervical Spine Pain*.

[4] Braun B. L. (1991). Postural differences between asymptomatic men and women and craniofacial pain patients. *Archives of Physical Medicine and Rehabilitation*.

[5] Swift T. R., Nichols F. T. (1984). The droopy shoulder syndrome. *Neurology*.

[6] Szeto G. P. Y., Straker L. M., O'Sullivan P. B. (2005). A comparison of symptomatic and asymptomatic office workers performing monotonous keyboard work - 2: Neck and shoulder kinematics. *Manual Therapy*.

[7] Andrade G. T., Azevedo D. C., Lorentz I. D. A. (2008). Influence of scapular position on cervical rotation range of motion. *Journal of Orthopaedic & Sports Physical Therapy*.

[8] Johnson G., Bogduk N., Nowitzke A., House D. (1994). Anatomy and actions of the trapezius muscle. *Clinical Biomechanics*.

[9] Ha S.-M., Kwon O.-Y., Yi C.-H., Jeon H.-S., Lee W.-H. (2011). Effects of passive correction of scapular position on pain, proprioception, and range of motion in neck-pain patients with bilateral scapular downward-rotation syndrome. *Manual Therapy*.

[10] Dvorák J. (1998). Epidemiology, physical examination, and neurodiagnostics. *The Spine Journal*.

[11] Owaydhah W. H., Alobaidy M. A., Alraddadi A. S., Soames R. W. (2017). Three-dimensional analysis of the proximal humeral and glenoid geometry using MicroScribe 3D digitizer. *Surgical and Radiologic Anatomy*.

[12] Hayasaki H., Martins R. P., Gandini L. G., Saitoh I., Nonaka K. (2005). A new way of analyzing occlusion 3 dimensionally. *American Journal of Orthodontics and Dentofacial Orthopedics*.

[13] Chen H., Lowe A. A., de Almeida F. R., Wong M., Fleetham J. A., Wang B. (2008). Three-dimensional computer-assisted study model analysis of long-term oral-appliance wear. Part 1: Methodology. *American Journal of Orthodontics and Dentofacial Orthopedics*.

[14] Boldt F., Weinzierl C., Hertrich K., Hirschfelder U. (2009). Comparison of the spatial landmark scatter of various 3D digitalization methods. *Journal of Orofacial Orthopedics*.

[15] Smith K., Hall T., Robinson K. (2008). The influence of age, gender, lifestyle factors and sub-clinical neck pain on the cervical flexion-rotation test and cervical range of motion. *Manual Therapy*.

[16] Van Dillen L. R., McDonnell M. K., Susco T. M., Sahrmann S. A. (2007). The immediate effect of passive scapular elevation on symptoms with active neck rotation in patients with neck pain. *The Clinical Journal of Pain*.

[17] Szeto G. P. Y., Straker L., Raine S. (2002). A field comparison of neck and shoulder postures in symptomatic and asymptomatic office workers. *Applied Ergonomics*.

[18] McDonnell M. K., Sahrmann S. A., Van Dillen L. (2005). A specific exercise program and modification of postural alignment for treatment of cervicogenic headache: a case report. *Journal of Orthopaedic & Sports Physical Therapy*.

[19] Sahrmann S. A. (2001). *Diagnosis and treatment of movement impairment syndromes*.

[20] Jull G., Sterling M., Falla D. (2008). *Whiplash, headache and neck pain: Research based directions for the physical therapies*.

[21] Mottram S. L., Woledge R. C., Morrissey D. (2009). Motion analysis study of a scapular orientation exercise and subjects' ability to learn the exercise. *Manual Therapy*.

[22] Hall T., Robinson K. (2004). The flexion-rotation test and active cervical mobility - A comparative measurement study in cervicogenic headache. *Manual Therapy*.

[23] Williams P. L. (1995). *Gray's Anatomy*.

[24] Hall T., Chan H. T., Christensen L., Odenthal B., Wells C., Robinson K. (2007). Efficacy of a C1-C2 self-sustained natural apophyseal glide (SNAG) in the management of cervicogenic headache. *Journal of Orthopaedic & Sports Physical Therapy*.

[25] Kim B.-B., Lee J.-H., Jeong H.-J., Cynn H.-S. (2016). Effects of suboccipital release with craniocervical flexion exercise on craniocervical alignment and extrinsic cervical muscle activity in subjects with forward head posture. *Journal of Electromyography & Kinesiology*.

[26] Jull G. A., Falla D., Vicenzino B., Hodges P. W. (2009). The effect of therapeutic exercise on activation of the deep cervical flexor muscles in people with chronic neck pain. *Manual Therapy*.

